# Proteomic analysis on effectors involved in BMP-2-induced osteogenic differentiation of beagle bone marrow mesenchymal stem cells

**DOI:** 10.1186/1477-5956-12-13

**Published:** 2014-03-01

**Authors:** Ji-Jie Hu, Ya-Wei Liu, Min-Yi He, Dan Jin, Hui Zhao, Bin Yu

**Affiliations:** 1 Department of Orthopaedics and Traumatology, Nanfang Hospital, Southern Medical University, Guangzhou, Guangdong 510515, China; 2 Department of Neurosurgery, Nanfang Hospital, Southern Medical University, Guangzhou, Guangdong 510515, China; 3 Department of Organ Transplantation, Zhujiang Hospital, Southern Medical University, Guangzhou, Guangdong 510282, China

**Keywords:** BMSCs, Osteogenic differentiation, rhBMP-2, Proteomics

## Abstract

**Objective:**

To identify the protein regulation profile of recombinant human bone morphogenetic protein-2 (rhBMP-2)-induced osteogenic differentiation in beagle bone marrow stem cells (BMSCs).

**Methods:**

Beagle BMSCs were isolated and cultured with or without rhBMP-2. Two-dimensional gel electrophoresis was used to determine the differences in protein expression in rhBMP-2-induced and non-induced BMSCs. Real-time PCR and western blotting analyses were used to verify the expression patterns of selected proteins.

**Results:**

After the induction, the osteogenic differentiation of beagle BMSCs was activated successfully. Nine and 11 proteins were found to be down- and up-regulated by rhBMP-2, respectively. The increase in Lim and SH3 domain protein 1(LASP1) and the decrease in ferritin were verified by real-time PCR and western blotting analyses.

**Conclusions:**

Among the 20 rhBMP-2-regulated factors, there is empirical evidence supporting the involvement of LASP1 and ferritin in osteogenic differentiation. LASP1 plays an important role in the regulation of the activity of the cytoskeleton, and ferritin is an important molecule in cellular iron homeostasis. Further studies focused on these 20 proteins will help elucidate the molecular mechanism(s) through which rhBMP-2 induces osteogenic differentiation of BMSCs.

## Introduction

The repair of large segmental bone defects caused by trauma or disease is a very difficult process in orthopedic clinics. Sources of autologous bone grafts are limited and allogeneic grafts may be rejected and might induce infection. The effect of artificial bone products for this purpose is still uncertain. Thus, the use of autologous bone marrow stem cells (BMSCs) to build/repair bone tissue and to treat bone defects has been proposed as a new treatment option in recent years. BMSCs are a subset of non-hematopoietic bone marrow stromal cells. They are easy to obtain and culture, and can be amplified *in vitro* and transformed to multi-line ages without loss of genetic stability [1]. BMSCs have a multi-potent differentiation potential *in vitro* under certain conditions. BMSCs can differentiate into adipocytes, osteoblasts, chondrocytes, and vascular smooth muscle, skeletal muscle, heart, endothelial, nerve, and liver cells, among others [2]. Thus, BMSCs have become the most promising seed cells for bone tissue engineering [3].

Bone morphogenetic proteins (BMPs) are bone growth factors that are known to induce osteogenesis [3]. These growth factors themselves are sufficient to induce bone formation [3]. Under certain conditions, they can induce undifferentiated mesenchymal cells to transform to bone cells and promote the proliferation of bone cells [3]. BMPs are currently considered the most important factor in osteogenesis [4,5]. The effect of recombinant human BMP-2 (rhBMP-2) on osteogenic differentiation of BMSCs is well-studied, and rhBMP-2 has already been applied widely in animal experiments and clinical applications [2]. In previous studies, we have also tested the utility of tissue-engineered bone containing rhBMP-2 and BMSCs in repairing bone defects in rabbits [6,7]. However, the molecular mechanisms of BMP-2-induced osteogenic differentiation are currently not known.

The source of BMSC specimens are mainly mice, rats, and humans. Human BMSCs are in limited supply and it is difficult to carry out studies with human BMSCs. BMSCs from mice or rats are quite different from human BMSCs in physiology. Beagle is considered an ideal large animal model widely used in pre-clinical toxicological experiments, basic medical research, radiation sickness treatment, and experimental surgery. Therefore, in this study, we established a model for BMSC primary cell culture and rhBMP-2-induced osteogenic differentiation using Beagle dogs. Proteomic analysis was used to identify proteins regulated by rhBMP-2. The results provide an improved understanding of the rhBMP-2-induced osteogenic differentiation of BMSCs.

## Material and methods

### Culture and induction of differentiation of BMSCs

According to standard bone marrow puncture procedures, 5 to 10 mL of bone marrow was aspirated from the posterior superior iliac spine of beagles. Heparin was added to the bone marrows to block coagulation and then the marrows were diluted with an equal volume of phosphate-buffered saline (PBS). The diluted bone marrows were layered onto lymphocyte separation medium (Sigma-Aldrich) at a ratio of 2:1, followed by centrifugation at 400 × *g* for 30 min. After centrifugation, the white layer between the two layers generated after centrifugation was collected and washed twice with PBS. Cells were seeded in culture plates at a density of 1 × 10^6^ cells/well and cultured in Dulbecco’s modified Eagle medium (DMEM) containing 10% fetal bovine serum, 100 U/mL penicillin, and 100 U/mL streptomycin at 37°C in an atmosphere of 5% CO_2_ and 95% humidity. After 72 h of incubation, the culture medium was changed once every 3 days. At the third generation, cells were trypsinized, and 3 × 10^6^ cells were plated in 100 mm culture dishes. The cells were stimulated with 20 mg/L rhBMP-2 (Hangzhou Jiuyuan Gene Engineering Co., Ltd., China) for one week, and the medium was changed once every three days. The morphology of the cells was observed everyday using an inverted phase-contrast microscope (IX81; Olympus, Japan). All protocols were approved by the Institutional Animal Care and Use Committee (IACUC) at Southern Medical University (Approval No. 120333, 114522).

### Gomori alkaline phosphatase staining

Gomori alkaline phosphatase staining was performed on cultured BMSCs as per the instructions of BCIP/NBT chromogenic alkaline phosphatase staining kit (Beyotime, China). After fixation, cells were washed with PBS three times, the BCIP/NBT staining working solution was added, and the cells were incubated at room temperature for 30 min. The BCIP/NBT staining working solution were then removed, and the cells were washed twice with distilled water to terminate the color reaction. The cells were observed and photographed using an inverted phase-contrast microscope (IX81; Olympus, Japan).

### Two-dimensional (2D) gel electrophoresis

Dry cells (100 mg) were lysed by adding 400 μL of lysis buffer (8 M urea, 4% CHAPS, 0.48% Tris base, 40 mM DTT, and 10 mMphenylmethylsulfonyl fluoride). The lysis mixture was incubated at room temperature for 60 min and centrifuged at 15,000 rpm for 60 min at 4°C. After centrifugation, the supernatant was collected and quantified with a 2D QUANT protein Assay Kit (GE, USA). The supernatant containing 300 μg of total cellular protein (about 20 μL) was mixed with the sample buffer (7 M urea, 2 M thiourea, 4% CHAPS, 65 mM DTT, 0.2% ampholytes, and a small amount of bromophenol blue) to a final volume of 450 μL. Two-dimensional gel electrophoresis (2-DE) was performed mainly according to the method described in a previous report [8] using a 24-cm pH4-7 linear DryStrip (GE, USA). After rehydration, is oelectric focusing (highest current set at 50 μA/gel, 20°C) and sodium dodecyl sulfate-polyacrylamide gel electrophoresis (SDS-PAGE) was performed. After electrophoresis, mass spectrometry-compatible silver staining was applied [9]. The gel was scanned using a UMAX Power Look 1100 transmission scanner to obtain images, and the images were analyzed with PDQuest 7.1.0 software. The analysis included the detection of protein spots, quantification, background removal, and cross-gel analysis. After comparison of the two gels, protein spots corresponding to a difference in expression greater than 2.5 times were selected for matrix-assisted laser desorption/ionization-time-of flight (MALDI-TOF) mass spectrometry.

### In-gel digestion and MALDI-TOF mass spectrometry

The protein spots were excised from gels into 1–2 mm^2^ blocks. After in-gel digestion, MALDI-TOF-TOF mass spectrometry was carried out using an ABI4700 (Applied Biosystems, USA) system. The obtained data were analyzed with the PMF Mascot database (http://www.matrixscience.com/). The parameters used were: species, mammal; trypsin digestion (the number of maximum missed cleavages was set to 1); carbamidomethylation of cysteine residues was set as a fixed modification; and mass error, 100 ppm.

### Real-time quantitative PCR

The RNA extracted from BMSCs (1 μg) was first reverse-transcribed into cDNA (PrimeScript; Takara, China). The cDNA was then used as the template for real-time quantitative PCR analysis (Maxima SYBR Green/ROX qPCR Master Mix; Thermo Scientific, Pittsburgh, PA). The primers for quantifying the mRNA expression of Lim and SH3 domain protein 1 (LASP1), ferritin light and heavy chains, and glyceraldehyde-3-phosphate dehydrogenase (GAPDH), which served as the internal control, are listed below (Table 1). The average ΔΔCT of LASP1, ferritin light and heavy chains, and GAPDH from samples analyzed in triplicate was calculated [10,11].

**Table 1:** Sequences of primers used in real-time quantitative PCR

**Gene**	**Gen bank accession no.**	**Primers**
GAPDH	NM_001003142.1	F: 5ʹ-ATCATCAGCAATGCCTCCTG-3ʹ
		R: 5ʹ-ATGGACTGTGGTCATGAGTC-3ʹ
LASP1	XM_859075.2	F: 5ʹ- GTGCGCTACAAGGAGGAGTT-3ʹ
		R: 5ʹ- CTCTGGAGTTCGGGTGTGTC-3ʹ
Ferritin light chain	NM_001024636.1	F: 5ʹ-AGGCCCTTTTGGATCTTCAT-3ʹ
		R: 5ʹ-CAGGTGGTCACCCATCTTCT-3ʹ
Ferritin heavy chain	NM_001003080.1	F: 5ʹ-CTGGAGCTCTACGCCTCCTA-3ʹ
		R: 5ʹ-TGGTTCTGCAGCTTCATCAG-3ʹ

### Western blotting

BMSCs in the exponential growth phase were lysed with radio-immunoprecipitation assay (RIPA) buffer. The expression level of LASP1 and ferritin light and heavy chains in BMSC lysates were analyzed by western blotting. The protein concentration of cell lysates was examined before resolving the total cellular protein (50 μg/lane) using SDS-PAGE. The proteins were transferred onto nitrocellulose membranes and the membranes were then blocked using 5% skim non-fat milk in Tris-buffered saline (TBS). The membranes were then incubated with primary antibodies specific for LASP1 (ab117806; Abcam, USA), ferritin light chain (ab69090; Abcam, USA), and ferritin heavy chain (ab65080; Abcam, USA). The membranes were washed three times in TBS containing 0.1% Tween 20 and then incubated with the appropriate secondary antibodies. The protein-antibody complexes were detected using a chemiluminescence reagent kit (ECL; Pierce Bio, USA). The protein-specific bands were analyzed by semi-quantitative densitometry.

### Statistical analysis

The data obtained from real-time quantitative PCR and the density of protein bands from western blotting analysis were analyzed with SPSS12.0 software (SPSS Inc., Chicago, IL). The data shown are the mean ± SD. The comparison of quantitative data was carried out using independent sample *t*-test and a value of *p* < 0.05 was considered to be statistically significant.

## Results

### rhBMP-induced osteogenesis in BMSCs

**Figure 1:**
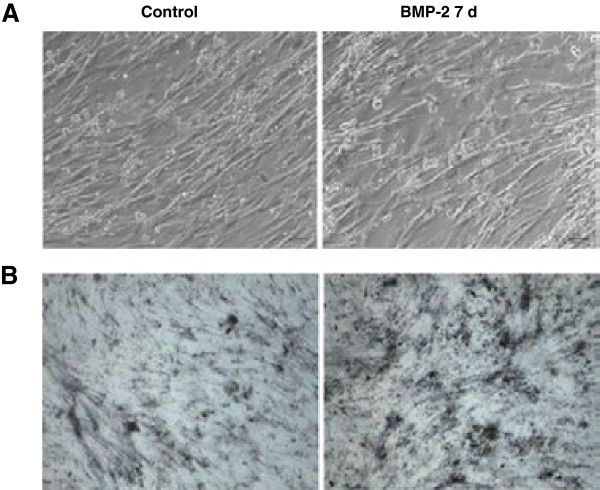
**rhBMP-2-induced BMSC osteogenic differentiation. (A)** Cell morphology of BMSCs without or with rhBMP-2 induction (labeled as control and BMP-2, respectively). The magnification is 100X. **(B)** Alkaline phosphatase staining was used to determine the status of osteogenic differentiation of BMSCs, without or with treatment with rhBMP-2. The image magnification is 100X.

The BMSCs were cultured and treated with rhBMP-2 as described in materials and methods. The cell morphology was analyzed by observation under a microscope. The non-induced cells were short, with spindle-like fusiform shapes. At day 7 of culture, the number of cells increased, the size of the cells enlarged, and the cells became elongated (Figure 1A, control). During continued culture, the cells remained long and spindle-shaped. After induction with rhBMP-2, the shapes of the BMSCs began changing gradually. Seven days after induction, the cell morphology drastically changed from long spindle-like cells to cubic cells (Figure 1A, BMP-2). The osteogenesis potential of rhBMP-2-induced BMSCs was evaluated by staining for alkaline phosphatase activity, a marker for ostoeblastic differentiation. When BMSCs were treated with rhBMP-2 for 7 days, a lot of dark blue dots appeared, indicating the presence of alkaline phosphatase activity (Figure 1B, BMP-2). In BMSCs that had not been treated with rhBMP-2, only a few alkaline phosphatase spots were detected (Figure 1B, control). These results show that BMSCs from beagles can be successfully cultured and that osteogenes can be induced by treatment with rhBMP-2.

### Two-dimensional gel electrophoresis

**Figure 2:**
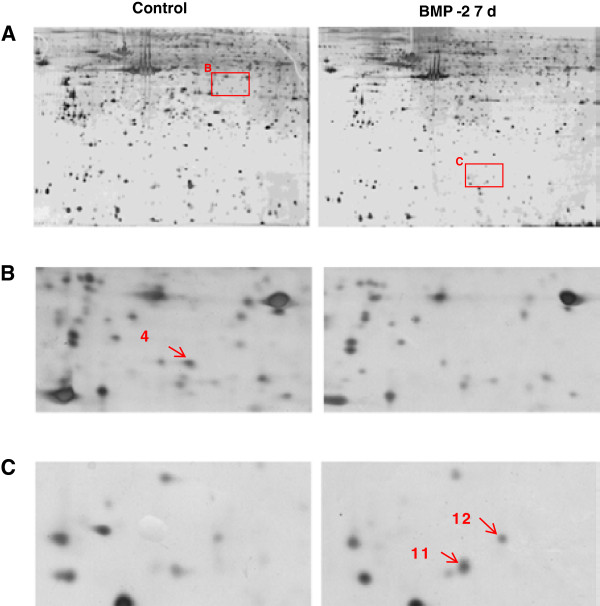
**Proteomics analysis of protein expression profiles in rhBMP-2-induced BMSCs. (A)** 2D gel electrophoresis maps of BMSCs without or with rhBMP-2 induction (labeled as control and BMP-2, respectively). **(B and C)** Close-up images of cropped sections of 2-DE maps shown in **(A)**. Protein number 4, LASP1; Number 11 and 12, ferritin light and heavy chains.

The protein extracts from non-induced and rhBMP-2 induced BMSCs were examined by two-dimensional gel electrophoresis followed by silver staining. The images of the silver-stained gels were clear and without background interference (Figure 2A, control and BMP-2). The protein spots were completely separated and similar results were obtained in all three independent trials of the experiment. An average of 1125 protein spots could be identified in the gel images from the three independent trials. The matching protein spots in the gels corresponding to the control (non-induced) and rhBMP-2-treated samples were considered to originate from the same proteins. The results showed that the percentage of matched proteins from the two groups of cells was approximately 90.84%.

**Figure 3:**
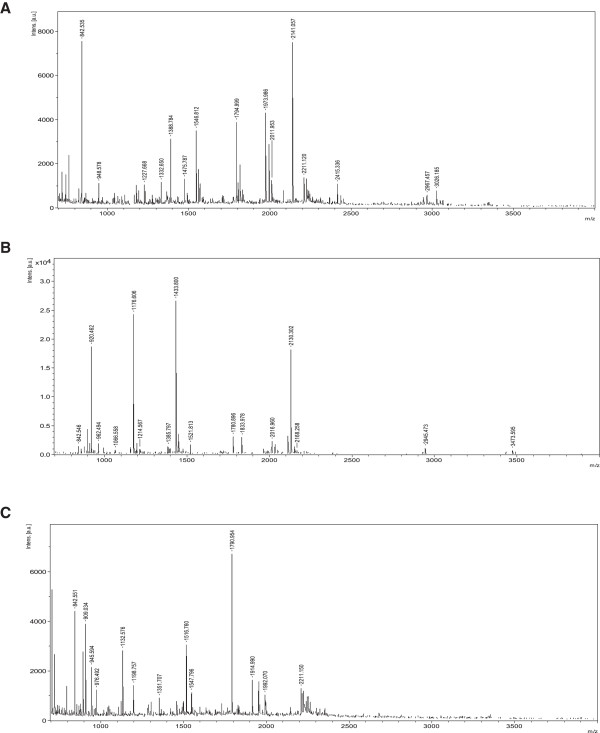
**Peptide fingerprint map obtained using MALDI-TOF mass spectrometry. (A)** LASP1. **(B)** Ferritin light chain. **(C)** Ferritin heavy chain.

**Figure 4:**
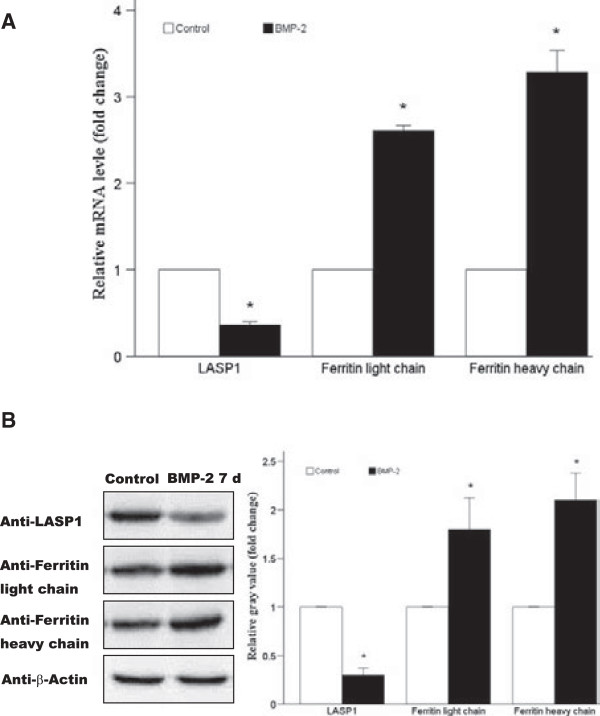
**Confirmation of RNA and protein expression levels by real-time quantitative PCR and western blotting analysis (n = 3, per treatment type). (A)** The expression of LASP1 and ferritin light and heavy chain mRNAs relative to that of GAPDH, the internal control, as detected by real-time PCR. **(B)** Expression of LASP1 and ferritin light and heavy chains, as assessed by western blotting analysis. The signal corresponding to the protein bands was quantified using densitometric scanning and the relative protein abundance was determined after normalization with the levels of β-actin protein. Data are presented as mean ± SD and compared using the *t*-test for independent samples. *p <0.05, indicating significant differences between the two samples compared.

After manual comparison and PDQuest 7.1.0 software analysis of the protein spots, 20 proteins with significant differences of expression levels between the control and induced cells were selected for further analysis. The differences were categorized as proteins that were up-or down-regulated by 2 times or more, and proteins that were either down-regulated (9 proteins) or up-regulated (11 proteins) in the BMP-2-induced cells were analyzed further by MALDI-TOF mass spectrometry for protein identification (Table 2). The proteins numbered as 4, 11, and 12 in Table 2 were identified as LASP1 isoform 2, ferritin light chain, and ferritin heavy chain (Figure 2B and C, marked by red arrows). Mass spectrometry peptide fingerprint peaks of these three proteins are shown in Figure 3A-C, respectively. The expression of the mRNAs corresponding to these three proteins in control and BMP-2-treated cells was examined by real-time quantitative PCR. Compared with the expression levels in the control cells, the expression of LASP1 decreased, while that of ferritin light and heavy chains increased, in the rhBMP-2-induced cells (*p* < 0.05) (Figure 4A). The expression levels of these three proteins in the non-induced and induced cells were also examined by western blotting. Consistent with the expression data for the mRNA, LASP1 protein levels were higher in control cells, while the levels of ferritin light and heavy chains was higher in the BMP-2-treated cells (Figure 4B). Densitometric analysis of protein bands from the western blots also indicated the same pattern of expression as that from the real-time quantitative PCR analysis of mRNA expression (*p* < 0.05) (Figure 4B).

**Table 2:** The proteins identified by proteomics analysis

	**Name**	**Accession number**	**Protein score**	**Mass**	**pI**	**Peptides identified**	**% Peptide coverage**	**rhBMP-2 induction/Control**
1	Eukaryotic translation initiation factor 3 subunit K isoform 1	gi|57036583	95	25342.6	4.81	YNPENLATLER	25.7	0.3
						ALTNLPHTDFTLCK		
						GIDRYNPENLATLER		
						CMIDQAHQEERPIR		
2	Heat shock protein beta-6	gi|57038366	100	17382.1	5.95	HFSPEEIAVK	21	<0.1
						VVGDHVEVHAR		
						HEERPDEHGYIAR		
3	Annexin A8 isoform 1	gi|73953207	102	36827.5	5.53	VFEEYER	39	<0.1
						FITILCTR		
						NLHSYFAER		
						ILVCLLQGSR		
						EGVIIEILASR		
						GIGTNEQAIIDVLTR		
						DLTETLQSELSGKFER		
						NLHSYFAERLYYALK		
						GSPHFNPEPDAEALYTAMK		
						AQFGKDLTETLQSELSGK		
4	LIM and SH3 domain protein 1 isoform 2	gi|73966152	89	30162.4	6.33	EPAAPVSIPR	38	<0.1
						QQSELQSQVR		
						IPYCNAHYPK		
						GFSVVADTPELQR		
						LKQQSELQSQVR		
						GKGFSVVADTPELQR		
						QSFTMVADTPENLR		
						TQDQISNIKYHEEFEK		
5	Glucose-6-phosphate isomerase	gi|296080693	94	63147	8.42	VDHQTGPIVWG	7.5	<0.1
						KTFTTQETIT		
						RSGDWKGYTGCETHAMLP YDQ		
6	RAD23 homolog B	gi|57094213	99	43171	4.77	A PTPVPALAPT	10.6	<0.1
						QAYFACEKNEN		
						AAKQEKPAEK		
						VVMVTKPKAVS		
7	Fibronectin 1	gi|345797318	99	262625	5.46	LEPGTEYTI	3	<0.1
						RARIAGYRLT		
						CSTTSNYEQDQ		
						STSQTTAPDAP		
						YVVGETWEKPYQG		
						LSISPSDNVVVLTNLL		
8	Poly(rC) binding protein 1	gi|222352151	95	37498	6.66	SSSPEVKGYWA	7	<0.1
						NSSMTNSTAASRPP		
9	Signal transducer and activator of transcription 1	gi|74005006	68	87335	5.74	FHAVEPYTK	6	<0.1
						LWYNMLVTE		
						RQQSACIGGPPN		
						VIDLETTSLPVVV		
10	Retinol-binding protein 4	gi|73998292	100	30177	8.48	QEELCLAR	49	>10
						RLPSVFHPGR		
						DPNGLPLEAQK		
						QRQEELCLAR		
						LIVHNGYCDGR		
						LIVHNGYCDGRSEPNTL		
						LLNLDGTCADSYSFVFSR		
						KDPEGLFLQDNIVAEFSVDENGR		
						GNDDHWIIDTDYDTYAVQYSCR		
11	Ferritin light chain	gi|66864897	100	20139.2	5.66	MGDHLTNLRR	73	4
						ELAEEKREGAER		
						DDVALEGVGHFFR		
						QNYSTEVEAAVNR		
						LATPQAGLGEYLFER		
						SLNQALLDLHALGSAR		
						ASYTYLSLGFYFDR		
						RLATPQAGLGEYLFER		
						ADPHLCDFLENHFLDEEVK		
12	Ferritin heavy chain	gi|302393573	100	21408.4	5.53	ELGDHVTNLR	66	3
						SVNQSLLELHK		
						YFLHQSHEER		
						IFLQDIKKPDR		
						SIKELGDHVTNLR		
						QNYHQDSEAAINR		
						SIKELGDHVTNLRK		
						YFLHQSHEEREHAEK		
						LATDKNDPHLCDFIETHYLNEQVK		
13	Annexin A4	gi|55742853	96	36075.2	5.72	VLVSLSAGGR	60	>10
						FLTVLCSR		
						NKSAYFAER		
						SDTSFMFQR		
						DEGNFLDDALMR		
						NHLLHVFDEYKR		
						NRNHLLHVFDEYK		
						SLYSFIKGDTSGDYR		
						AEIDMMDIRESFKR		
						SLYSFIKGDTSGDYRK		
						NRNHLLHVFDEYKR		
						DLMDDLKSELSGNFER		
						GGTVKPASGFSATEDAQTLR		
						VLVSLSAGGRDEGNFLDDALMR		
14	Proteasome activator complex subunit 3, isoform 6	gi|73965646	84	29601.6	5.69	YPHVEDYR	45	3
						LKVDSFRER		
						YPHVEDYRR		
						IAKYPHVEDYRR		
						RLDECEEAFQGTK		
						TVESEAASYLDQISR		
						ITSEAEDLVANFFPK		
						TVTEIDEKEYISLR		
						IEDGNNFGVSIQEETVAELR		
15	Adenosylhomocysteinase isoform 1	gi|73991635	100	48260.4	5.98	RIILLAEGR	14.8	3
						FDNLYGCR		
						YPQLLSGIR		
						VNIKPQVDR		
						SKFDNLYGCR		
						VAVVAGYGDVGKGCAQALR		
16	Histidyl-tRNA synthetase, cytoplasmic, isoform 1	gi|73949318	73	57880.3	5.44	IFSIVEQR	8	>10
						YDLTVPFAR		
						DQGGELLSLR		
						KVPCVGLSIGVER		
17	Solute carrier family 25 (mitochondrial carrier; phosphate carrier), member 3	gi|119618005	87	40,095	9.45	SVVSVLNKE	10	4
						DAAPKMYKEEGL		
						IFNGFSVTLKEDGVRG		
18	ATP-binding cassette, sub-family B (MDR/TAP), member 1	gi| 51094928	101	141479	9.06	EGLMPNTL	6	3
						LSLEVKKG		
						AVKEANAYDFI		
						GLQPAFAIIFSKIIG		
						LTLVILAISPVLGLS		
						AGNLEDLMSNITNRSDIND		
19	DEAD (Asp-Glu-Ala-Asp) box helicase 1	gi| 148666036	66	82432	6.80	ERLGKNHI	7	>4
						CLHGDRKPHERKQN		
						GCYLDIDKGHVKFSK		
						AIGSDGLCCQSREVKE		
20	Isocitrate dehydrogenase 1 (NADP+), soluble	gi|119590847	99	46659	6.53	EVSIETIEA	11	3
						SPNGTIRNI		
						GTQKVTYLVHNF		
						DDMVAQAMKSEGGFI		
21	Filamin B, beta	gi| 410336835	73	278164	5.47	LEFLDRES	2.5	3
						EAGEGDVSV		
						PHVVKVFFAG		
						ECSDNGDGTCS		
						ILVKYNDKHIPGS		
						KQPAKFTVDTISAG		

## Discussion

In this study, beagle BMSCs were successfully isolated and cultured. The BMSCs were stimulated with rhBMP-2 to induce osteogenic differentiation. After 7 days of stimulation, osteogenic differentiation of BMSCs was seen, confirmed by the morphological changes in BMSCs and the appearance of alkaline phosphatase activity, a marker for osteoblastic differentiation. The protein expression profiles of untreated control and rhBMP-2-treated BMSCs were characterized using 2D gel electrophoresis. Comparison of the two protein expression profiles indicated that 9 proteins were up-regulated and 11 proteins were down-regulated after stimulation of BMSCs with rhBMP-2. Based on previously published reports and bioinformatics analysis, two proteins, LASP1 and ferritin (light and heavy chains), were selected for further analysis as candidate proteins involved in osteogenic differentiation induced by BMP-2. The results from real-time quantitative PCR and western blotting analyses confirmed the down-regulation of LASP1 and the up-regulation of ferritin in rhBMP-2-induced BMSCs.

LASP1 protein contains one LIM, one SH3, and two actin domains, and was first discovered in 1996 as a protein that is over-expressed in breast cancer [12]. Studies about LASP1 have almost all concentrated on LASP1 expression in tumors. LASP1 is involved in regulating cellular functions associated with actin-cytoskeleton-associated membrane rearrangements [13-15]. One study showed that aberrant expression of LASP1 can affect the formation of cartilage tissue [16]. To our knowledge, ours is the first study demonstrating a decrease in LASP1 expression during the process of rhBMP-2-induced osteogenic differentiation.

Ferritin is an important molecule in iron homeostasis in cells [17]. Iron overload can inhibit osteoblast differentiation and induce ferritin expression. Iron-provoked inhibition of osteoblast activity is mediated by ferritin and its ferroxidase activity [18]. We found that the expression of ferritin light and heavy chains was increased in BMSCs after induction of differentiation by rhBMP-2. The possible role for the up-regulation of ferritin might be that when stem cells in bone marrow are stimulated by rhBMP-2, over-expression of ferritin causes the enrichment of iron, thereby inhibiting osteogenic differentiation. However, the concentration of iron ions in the culture medium is maintained at steady levels, hence osteogenic differentiation is not inhibited under *in vitro* culture conditions, even though ferritin expressed is increased upon stimulation with rhBMP-2. The extent and exact mechanism(s) through which the expression of LASP1 and ferritin are altered upon stimulation with BMP-2 need to be addressed in further mechanistic studies of osteogenic differentiation of BMSCs.

In summary, rhBMP-2 induces osteogenic differentiation of BMSCs, decreases LASP1 expression, and increases ferritin expression. LASP1 is a key factor involved in the regulation of the cytoskeleton and ferritin controls cellular iron homeostasis, and the expression changes in these proteins likely play an important role in the process of rhBMP-2-induced osteogenic differentiation. The results of this study provide additional information regarding the role of changes in expression of specific proteins in driving BMP-2-induced osteogenic differentiation and indicate the need for further studies to elucidate the specific mechanism(s) through which BMP-2 promotes differentiation of BMSCs.

## Competing interests

The authors declare that they have no competing interests.

## Authors’ contributions

JH carried out the molecular genetic studies, participated in the sequence alignment and drafted the manuscript. MH carried out the immunoassays. DJ participated in the sequence alignment. HZ participated in the design of the study and performed the statistical analysis. YL conceived of the study, and participated in its design and coordination and helped to draft the manuscript. BY participated in the molecular genetic studies and the sequence alignment. All authors read and approved the final manuscript.

## References

[B1] Liu W Liu M Zhu JS Hao HW Dong NZ Zhou HB *In vitro* culture, identification and osteogenic differentiation of human bone marrow mesenchymal stem cells Chinese J Tissue Eng Res 2012 16 14 2515 2519

[B2] Pontikoglou C Deschaseaux F Sensebé L Papadaki HA Bone marrow mesenchymal stem cells: biological properties and their role in hematopoiesis and hematopoietic stem cell transplantation Stem Cell Rev 2011 7 3 569 589 10.1007/s12015-011-9228-8 21249477

[B3] Potier E Noailly J Ito K Directing bone marrow-derived stromal cell function with mechanics J Biomech 2010 43 5 807 817 10.1016/j.jbiomech.2009.11.019 19962149

[B4] Bramono DS Murali S Rai B Ling L Poh WT Lim ZX Stein GS Nurcombe V van Wijnen AJ Cool SM Bone marrow-derived heparan sulfate potentiates the osteogenic activity of bone morphogenetic protein-2 (BMP-2) Bone 2012 50 4 954 964 10.1016/j.bone.2011.12.013 22227436 PMC3589980

[B5] Rui YF Lui PP Lee YW Chan KM Higher BMP receptor expression and BMP-2-induced osteogenic differentiation in tendon-derived stem cells compared with bone-marrow-derived mesenchymal stem cells Int Orthop 2012 36 5 1099 1107 10.1007/s00264-011-1417-1 22134708 PMC3337107

[B6] Hu JJ Jin D Quan DP Zhong SZ Chen JH Wei KH Zhao J Pei GX Bone defect repair with a new tissue-engineered bone carrying bone morphogenetic protein in rabbits J First Mil Med Univ 2005 25 11 1369 1374 16305958

[B7] Hu JJ Jin D Quan DP Wei KH Chen JH Zhong SZ Pei GX The dose-effect correlation between tissue engineered bone loaded BMP at different densities and new bone formation *in vivo* Chinese J Orthopaedics 2006 26 3 196 201

[B8] Liu Y Song F Wu WK He M Zhao L Sun X Li H Jiang Y Yang Y Peng K Triptolide inhibits colon cancer cell proliferation and induces cleavage and translocation of 14-3-3 epsilon Cell Biochem Funct 2012 30 4 271 278 10.1002/cbf.2793 22315045

[B9] Liu Y He M Sun X Peng K Zhao L Alteration of nuclear protein profiling for NIH-3T3 cells exposed to H2O2 Cell Biochem Funct 2010 28 7 578 584 10.1002/cbf.1693 20941747

[B10] Schmittgen TD Livak KJ Analyzing real-time PCR data by the comparative CT method Nat Protoc 2008 3 1101 1108 10.1038/nprot.2008.73 18546601

[B11] Livak KJ Schmittgen DT Analysis of relative gene expression data using real-time quantitative PCR and the 2-ΔΔCt method Methods 2001 25 402 408 10.1006/meth.2001.1262 11846609

[B12] Tomasetto C Moog-Lutz C Régnier CH Schreiber V Basset P Rio MC Lasp-1 (MLN 50) defines a new LIM protein subfamily characterized by the association of LIM and SH3 domains FEBS Lett 1995 373 3 245 249 10.1016/0014-5793(95)01040-L 7589475

[B13] Chew CS Chen X Parente JA Jr Tarrer S Okamoto C Qin HY Lasp-1 binds to non-muscle F-actin *in vitro* and is localized within multiple sites of dynamic actin assembly *in vivo* J Cell Sci 2002 115 4787 4799 10.1242/jcs.00174 12432067

[B14] Schreiber V Moog-Lutz C Regnier CH Chenard MP Boeuf H Vonesch JL Tomasetto C Rio MC Lasp-1, a novel type of actin-binding protein accumulating in cell membrane extensions Mol Med 1998 4 675 687 9848085 PMC2230251

[B15] Zhang H Chen X Bollag WB Bollag RJ Sheehan DJ Chew CS Lasp1 gene disruption is linked to enhanced cell migration and tumor formation Physiol Genomics 2009 38 3 372 385 10.1152/physiolgenomics.00048.2009 19531578 PMC3774566

[B16] Hermann-Kleiter N Ghaffari-Tabrizi N Blumer MJ Schwarzer C Mazur MA Artner I Lasp1 misexpression influences chondrocyte differentiation in the vertebral column Int J Dev Biol 2009 53 7 983 991 10.1387/ijdb.072435nh 19378260

[B17] Yang Q Jian J Abramson SB Huang X Inhibitory effects of iron on bone morphogenetic protein 2-induced osteoblast ogenesis J Bone Miner Res 2011 26 6 1188 1196 10.1002/jbmr.337 21308772

[B18] Zarjou A Jeney V Arosio P Poli M Zavaczki E Balla G Balla J Ferritin ferroxidase activity: a potent inhibitor of osteogenesis J Bone Miner Res 2010 25 1 164 172 10.1359/jbmr.091002 19821764

